# Frequency of *Chlamydia trachomatis *in *Ureaplasma*-positive healthy women attending their first prenatal visit in a community hospital in Sapporo, Japan

**DOI:** 10.1186/1471-2334-12-82

**Published:** 2012-04-02

**Authors:** Tomohiro Yamazaki, Megumi Matsumoto, Junji Matsuo, Kiyotaka Abe, Kunihiro Minami, Hiroyuki Yamaguchi

**Affiliations:** 1 Department of Medical Laboratory Science, Faculty of Health Sciences, Hokkaido University, Nishi-5 Kita-12 Jo, Kita-ku, Sapporo, Hokkaido 060-0812, Japan; 2 Toho Obstetrics and Gynecology Hospital, Higashi-15, Kita-17 Jo, Higashi-ku, Sapporo 065-0017, Japan

**Keywords:** Chlamydia trachomatis, Ureaplasma urealyticum, Ureaplasma parvum, Mixed infection, PCR

## Abstract

**Background:**

Although *Chlamydia trachomatis *is the most commonly reported pathogen that causes urogenital infection such as urethritis or cervicitis, *Ureaplasma parvum *and *Ureaplasma urealyticum*, which are commensals in the genital tract, have also now been recognized as contributors to urogenital infection. However, whether the presence of either *U. parvum *or *U. urealyticum *is related to that of *C. trachomatis *in the urogenital tract remains unknown. We therefore attempted to estimate by PCR the prevalence of *C. trachomatis, U. parvum *and *U. urealyticum *in endocervical samples obtained from healthy women attending their first prenatal visit in Sapporo, Japan.

**Methods:**

The samples were taken from 303 apparently healthy women, and the extracted DNAs (*n *= 280) were used for PCR detection targeting *C. trachomatis, U. parvum *and *U. urealyticum*. Statistical analysis of the data was performed by Fisher's exact test.

**Results:**

PCR detection revealed that the prevalence of *C. trachomatis, U. parvum *and *U. urealyticum *was 14.3% (40/280), 41.7% (117/280) and 8.9% (25/280), respectively. *C. trachomatis ompA *genotype D was most frequently identified. Surprisingly, either *C. trachomatis *or *Ureaplasma *spp. was detected in almost half of the healthy women. Mixed infection of *C. trachomatis *with either *U. parvum *or *U. urealyticum *was also observed in 9.2% (26/280) of the women. There was a significant association between *C. trachomatis *and either *U. parvum *(*p *= 0.023) or *Ureaplasma *total (*p *= 0.013), but not *U. urealyticum *(*p *= 0.275).

**Conclusion:**

This study demonstrated that the presence of *Ureaplasma *had a significant effect on the presence of *C. trachomatis *in the genital tract of healthy women, suggesting that mixed infection is an important factor in bacterial pathogenesis in the genital tract.

## Background

Urogenital tract infections are a major cause of morbidity in sexually active individuals worldwide, therefore, the World Health Organization has stated that sexually transmitted diseases (STDs) rank second in importance after cancer as treatable diseases in women. In particular, *Chlamydia trachomatis *is the leading cause of bacterial STD, with an estimated 5 million new cases annually worldwide [1-3]. *C. trachomatis *infection can cause testicular atrophy, epididymitis and orchitis in men, and ductal obstruction, pelvic inflammatory disease, tubal occlusion and extrauterine pregnancy in women [4-9]. However, the aetiology of most cases of chlamydial infection is undetermined and it could be multifactorial in nature, because of complications with commensal bacteria or mixed infections with other pathogens [10-12]. Therefore, the prevalence of *C. trachomatis *and other pathogens needs to be investigated.

*Ureaplasma *are currently separated into two species: *Ureaplasma urealyticum *and *Ureaplasma parvum*, which are both thought to be genital tract commensals [13-17]. They are commonly found in healthy persons, therefore, their pathogenic role can be difficult to prove in a small population of individuals. Meanwhile, several studies have reported that *Ureaplasma *are associated with some diseases including non-gonococcal urethritis, pregnancy complications and prenatal infections, more often than are normal flora [18-20]. Thus, it might be that *Ureaplasma *perturb homeostasis in the genital tract, which provides a survival advantage for *C. trachomatis*. However, data regarding mixed infection of *C. trachomatis *with *Ureaplasma *are limited [13-15].

In the present study, we therefore attempted to estimate by PCR and culture (*C. trachomatis*, inclusion forming assay; *Ureaplasma*, urease test), the prevalence of *C. trachomatis, U. parvum *and *U. urealyticum *in healthy women attending their first prenatal visit at a community hospital in Sapporo, Japan.

## Methods

### Bacteria

*C. trachomatis *D/UW3 Cx strain (VR-855) and *U. parvum *(ATCC-27813) were purchased from ATCC (Manassas, VA, USA). *C. trachomatis *and *U. parvum *were propagated in the HEp-2 cell culture system [21] and PPLO medium [1.5% (w/v) PPLO powder, 20% (v/v) horse serum, 5% (w/v) yeast extract, 1% (w/v) urea, 0.1% (w/v) phenol red, antibiotics (10 μg/ml vancomycin; 1 μg/ml amphotericin B), pH 6.0], respectively. The numbers of infectious progenies for *C. trachomatis *were determined as inclusion forming units (IFU) by counting chlamydial inclusions formed in HEp-2 cells using fluorescein isothiocyanate (FITC)-conjugated monoclonal anti-*Chlamydia *antibody specific to *Chlamydia *lipopolysaccharide (LPS) (Denka Seiken Co. Ltd., Tokyo, Japan) [21]. The numbers *U. parvum *were also determined as colony-forming units (CFU) by counting colonies formed on the PPLO agar under a phase-contrast microscope.

### Endocervical samples

Three hundred and three samples were obtained from apparently healthy women attending their first prenatal visit at Toho Obstetrics and Gynecology Hospital [number of deliveries, 1,332 per year (2010); number of caesarean sections, 310 per year (2010); number of vacuum extractions, 106 per year (2010)], located on the outskirts of Sapporo City, Japan, from July 2010 to September 2010. The average age (± SD) of healthy women attending this hospital was 28.28 ± 5.25 years, and the age distribution was as follows: 94 (20-24 years), 93 (25-29 years), 59 (30-34 years), 25 (35-40 years), and nine (> 40 years). The samples were collected by scraping the endocervix of each woman with a sterile cotton applicator. The applicator was immediately immersed and resuspended in 1 ml sucrose-phosphate-glutamic acid buffer [SPG: 0.2 M sucrose, 3.8 mM KH_2_PO_4_, 6.7 mM Na_2_HPO_4_, 5 mM L-glutamic acid (pH 7.4)][21], and stored at -80°C until use.

### Ethics

Written informed consent was obtained from all women, and the study was approved by the ethics committees of the Faculty of Health Sciences, Hokkaido University and Toho Obstetrics and Gynecology Hospital.

### DNA extraction

One hundred microlitres of SPG solution in each sample was used for DNA extraction using a QIAmp DNA mini kit (Qiagen, Valencia, CA, USA), according to manufacturer's instructions. The solution was centrifuged at 18,900 × g for 30 min. Pellets were then used for DNA extraction. The DNA was eluted in 50 μl of the elution buffer supplied with the kit, quantitated spectrophotometrically and stored at -20°C until use.

### PCR detection and lineage analysis with *ompA *full sequences

Table 1 shows primer sets and conditions for PCR amplification of *C. trachomatis *[22], *U. parvum *[13], *U. urealyticum *[13], and most bacteria [23]. The quality of extracted DNA was confirmed by PCR amplification using universal primers that target bacterial *16S rRNA*, which is conserved across a broad spectrum of bacteria. DNA samples that resulted in negative PCR for bacterial *16S rRNA *were discarded. Finally, the samples (*n *= 280) that yielded PCR products of the expected size were used for PCR amplification of *C. trachomatis *(target gene, *ompA*) [22], *U. parvum *[target gene, 5' ends of the multiple-banded antigen gene (MBA) with upstream region] [13] and *U. urealyticum *(target gene, MBA with upstream region) [13]. Template DNA (2 μl) (average amount of DNA per sample: 63.64 ± 89.16 ng/μl) was used for each PCR. Reactions were carried out in 25 μl reaction buffer [each dNTP, 200 μmol; 1× commercial reaction buffer (New England Biolabs, Herts, UK)] containing *Taq *DNA polymerase [0.625 U *Taq *DNA polymerase (New England Biolabs). The PCR cycle consisted of 10 min denaturation at 94°C followed by 30-45 cycles, each of 30 s denaturation at 94°C; 30 s of annealing at 52-58°C; and 45 s of extension at 72°C. The amplified products were separated by 1.2% agarose gel electrophoresis and visualized by ethidium bromide staining. Each PCR was performed at least two times, for confirmation of PCR specificity and reproducibility. In addition, to prevent contamination, the preparation of the PCR mixture was performed in a separate room. All products of *ompA *amplified in *C. trachomatis*-positive specimens were fully sequenced (Macrogen, Seoul, Korea) and each of the genotypes was determined through BLAST search. Lineage analysis with *ompA *sequences was also performed by the following method: *C. trachomatis ompA *sequences were aligned with MUSCLE software [23]. Then, the phylogenetic tree was constructed using the neighbour-joining method with MEGA5 software [24]. The following reference sequences were used: A/Sa1 (M58938), B/TW-5 (M17342), C/TW3 (M17343), D/B-120 (X62918), E/Bour (X52557), F/IC-Cal3 (X52080), G/UW57 (AF063199), H/Wash (X16007), I/UW-12 (AF063200), J/UW36 (AF063202), K/UW31 (AF063204), L1/440 (M36533), L2/434 (M14738), and L3/404 (X55700). *Chlamydia muridarum *MoPn (M64171) was also used as an out-group sequence. The gene accession numbers of sequences identified in this study were deposited in "DNA Data Bank of Japan" and available from authors upon request.

**Table 1:** Primer sequences and PCR conditions used for this study

Microorganism	Target gene	Primer	Sequence	Annealing condition;	Expected	Reference [Reference number]
				
				Temperature/cycle	fragment size	
Most bacteria	*16S rRNA*	Forward (Bac3)	5'-AGA GTT TGA TYM TGG CTC AG-3'*	52/35	nearly full-length	Horn et al., 1999 [22]
		
		Reverse (Bac4)	5'-CAK AAA GGA GGT CC-3'**			

*Chlamydia trachomatis*	*ompA*	Forward (CtOmp1)	5'-ATG AAA AAA CTC TTG AAA TCG G-3'	55/35	1,100 bp	Jurstrand et al., 2010 [23]
		
		Reverse (CtOmp2)	5'-ACT GTA ACT GCG TAT TTG TCT G-3'			

*Ureaplasma parvum*	MBA gene with	Forward (UMS-57)	5'-(T/C)AA ATC TTA GTG TTC ATA TTT TTT AC-3'	58/35	326/327 bp	Kong et al., 2000 [13]
	
	it upstream region***	Reverse (UMA222)	5'-GTA AGT GCA GCA TTA AAT TCA ATG-3'			

*Ureaplasma urealyticum*	MBA gene with it upstream region	Forward (UMS-170)	5'-GTA TTT GCA ATC TTT ATA TGT TTT CG-3'	58/45	476 bp	Kong et al., 2000 [13]
		
		Reverse (UMA263)	5'-TTT GTT GTT GCG TTT TCT-3'			

*Y, C or T; M, A or C

**K, G or T

***MBA, 5' ends of the multiple-banded antigen gene

### PCR detection limit for *C. trachomatis *and ureaplasmas

To determine the detection limit of PCR for *C. trachomatis *and *Ureaplasma *spp., spike experiments were performed. *U. parvum *was used as a representative *Ureaplasma*. Several sets of 100 μl of SPG solution in the pooled swab samples, which proved negative for *C. trachomatis *and *Ureaplasma*, were prepared. The sets were spiked with serial dilutions of either *C. trachomatis *VR-855 from 10^-2 ^to 10^4 ^IFU or *U. parvum *ATCC-27813 from 10^-1 ^to 10^5 ^CFU per sample. The DNA extraction of spiked samples, as well as from clinical specimen, was used for the PCR-detection method with primer sets targeting to *C. trachomatis ompA *and *Ureaplasma *MBA as described above.

### Biological detections

The presence of infectious *C. trachomatis *in the samples confirmed as *C. trachomatis*-PCR positive was determined by inclusion formation assay on HEp-2 cells. The presence of viable *Ureaplasma *in the samples confirmed as either *U. parvum *or *U. urealyticum *PCR-positive was also determined using a urease assay as follows. In brief, a sample solution of 5 μl was diluted to 100 μl with SPG, and then passed through a filter with a 0.22-μm pore size. The filtrate was cultured in a total of 200 μl of PPLO medium at 37°C for 7 days. Samples that turned from yellow to red after the incubation were considered *Ureaplasma *positive.

### Statistical analysis

Comparison between the frequency of *C. trachomatis *and that of *U. parvum *or *U. urealyticum *was done by Fisher's exact test (two-way ANOVA; Statview, Abacus Concepts Inc., Piscatway, NJ, USA). A *p *value < 0.05 was considered significant.

## Results and discussion

### Quality control of DNA extracted from endocervical samples

We assessed by PCR amplification the bacterial flora in the genital tract, to confirm whether extracted DNA was suitable for PCR amplification that target *C. trachomatis *or *Ureaplasma *spp. Three hundred and three samples were individually assessed by PCR with a primer set targeting bacterial *16S rRNA*. The average amount of DNA was 63.64 ± 89.16 ng/μl, which indicated successful DNA extraction, therefore, it was expected that all samples would be available for PCR. However, in 23 samples (9.2%) PCR failed to amplify 16S rRNA gene, even though the amount of extracted DNA was never low. Therefore, the 23 samples without amplification were omitted and the remaining 280 were used for this study. Standard PCR techniques, which can be applied to analyse the entire microorganism community of complex biological samples obtained from living individuals, has been universally used. However, it is well known that haemoglobin, lactoferrin, heparin, and bile acids, which are ubiquitous to environments such as the genital tract, inhibit PCR amplification [25]. This suggests that it is absolutely necessary to check the DNA quality carefully to confirm successful PCR amplification from genital swab samples.

### PCR detection of *C. trachomatis, U. parvum *and *U. urealyticum*

**Figure 1:**
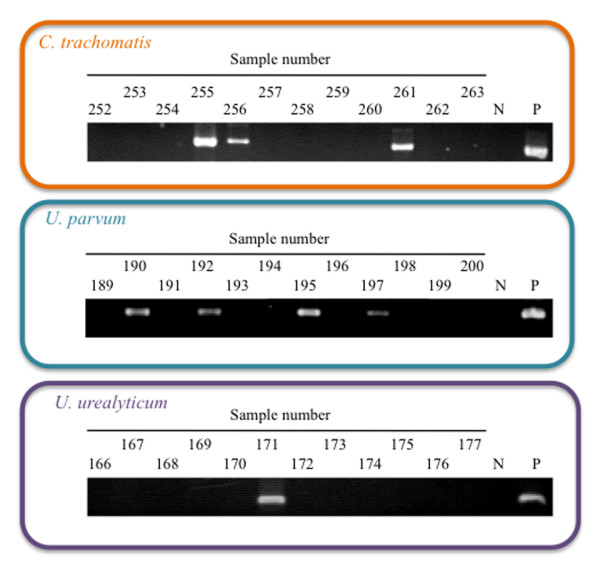
**Representative PCR detection images showing *C. trachomatis, U. parvum *and *U. urealyticum *amplification**. N, negative control (molecular grade water); P, positive control (DNA extracted from each of the bacteria).

The detection limit of the PCR for either *C. trachomatis ompA *or *Ureaplasma *MBA with upstream region was examined by using DNAs extracted from the pooled genital swabs [confirmed that all targeted bacteria (*C. trachomatis *and *Ureaplasma *spp.) were negative] that had been spiked with defined numbers of either *C. trachomatis *or *U. parvum*. The detection limit of the PCR in spiked genital swabs for *C. trachomatis *and *U. parvum *(as a representative *Ureaplasma*) was 1 IFU and 100 CFU per 100 μl of swab sample, respectively. The spiked samples revealed that the PCR system allowed stable and sensitive detection of each target DNA. Although the PCR primers that targeted *C. trachomatis ompA *for *C. trachomatis *detection were used for this study, it has also been shown worldwide that cryptic plasmid gene permits detection of *C. trachomatis *from clinical specimens [26-28]. However, so far it has also been reported that there is absence, variation or deletion of the plasmid in *C. trachomatis *[29-31], in particular, causing a clinical problem with false-negative results for *C. trachomatis *in Europe [29-31]. Although plasmid copy number is generally much higher than that of a genomic gene, which increases the sensitivity of PCR detection, we did not use cryptic plasmid for PCR targeting of *C. trachomatis *detection to prevent false-negative results. Detection of *U. parvum *and *U. urealyticum *with PCR primers targeting MBA genes with variable regions has been well documented for *Ureaplasma *detection [13,32-36] therefore, we selected this gene for PCR detection of *Ureaplasma *spp. In fact, as shown in Figure 1 (representative PCR results), each of the PCR products was clearly visible, permitting us to make accurate judgements.

### Prevalence of *C. trachomatis, U. parvum *and *U. urealyticum*

**Figure 2:**
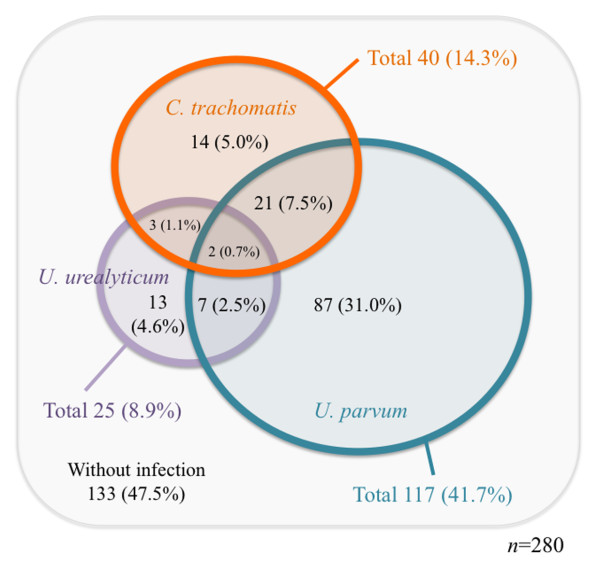
**Detection frequencies of *C. trachomatis, U. parvum *and *U. urealyticum *in DNA extracted from genital swabs**.

As shown in Figure 2, PCR detection revealed that the prevalence of *C. trachomatis, U. parvum *and *U. urealyticum *was 14.3%, 41.7% and 8.9%, respectively. The prevalence of *C. trachomatis *in healthy women has been estimated worldwide. The detection frequency among healthy women varies between countries and cities (46% Colombia [37]; 22.9% England [38]; 3.9% USA (Baltimore) [39]; 0.2% Australia [40]), probably depending on prevailing environmental factors and population living habits. However, its infectious frequency is believed to be around 10% on an average from a global perspective [1,2]. As compared to other studies with healthy women in Japan (~5.8% on average; 3.0-3.8% Tokyo [39,41]; 5.6% Fukuoka [42]; 8.3-8.8% Miyasaki [43,44]; 1.7% Nasushiobara [45]; 9.5% Wako [46]), the prevalence of *C. trachomatis *in Sapporo City that we estimated (14.3%) was unique. Our result is supported by a previous study showing a *C. trachomatis *detection frequency of 11.3% in Sapporo [47]. Thus, the prevalence of *C. trachomatis *in Sapporo, Japan, is likely to be high. Although we cannot explain the exact reason for this high prevalence, it may be related to the fact that the city has a relatively large population (~2 million) with an active nightlife, (Susukino district, ~3.7 km from the hospital), which particularly attracts sexually active young people. We also found that the detection frequency in the youngest age group (20-24 years), who were more sexually active, predictably increased from 14.3% to 23.4%.

PCR detection also revealed that the prevalence of *U. parvum *and *U. urealyticum *was 41.7% and 8.9%, respectively. Other studies with healthy women also have reported that the detection frequency of *U. parvum *and *U. urealyticum *was estimated at ~50% (57 and 87% Australia [13,48]; 52% Japan [49]; 33.2 and 86.8% China [50]; 86% Italy [51]; 17.9% Poland [52]) and 10% (6.1-19% Australia [13]; 8.7% Japan [49]; 4.6-10.5% China [50]; 14% Italy [51]; 2.6% Poland [52]). The findings with our data suggested that the distribution of *Ureaplasma *is spreading worldwide and that the species are commonly found in healthy people as presumably commensal bacteria, therefore, their pathogenic role would be minimal [53-56]. Nevertheless, it has been increasingly reported that *Ureaplasma *spp. are associated with non-gonococcal urethritis, chorioamnionitis, preterm birth, perinatal morbidity, and mortality, more often than are normal flora [53-55]. Thus, our knowledge regarding the pathogenesis of *Ureaplasma *still remains paradoxical and is limited, suggesting that further, larger epidemiological studies with healthy people and patients with urogenital disorders are needed.

The gold standard for bacterial detection from clinical specimens is probably culture; therefore, we also assessed whether *C. trachomatis *and *Ureaplasma *spp. in PCR-positive samples could be detected by using biological detection systems, IFU assay and urease assay, respectively. However, contrary to our expectation, the detection frequencies of *C. trachomatis *and *Ureaplasma *spp. in PCR-positive samples decreased to 30% and 2%, respectively. Although it is necessary to clarify the exact reason, it is possible that freeze-thawing of samples crucially caused a decrease in detection frequencies.

### Mixed infection of *C. trachomatis *and *Ureaplasma*

Surprisingly, either *C. trachomatis *or *Ureaplasma *spp. was detected in almost half of the healthy women (52.5%, 147/280), and mixed infection with *C. trachomatis *and either *U. parvum *or *U. urealyticum *was observed in 9.2% (26/280) (Figure 2, See overlapping area). It is intriguing that there was a significant association between *C. trachomatis *and either *U. parvum *(*p *= 0.023) or *Ureaplasma *total (*p *= 0.013), but not *U. urealyticum *(*p *= 0.275) (Table 2). Meanwhile, several studies also estimated the mixed infection frequency; however, correlation between the frequency of *C. trachomatis *and either *U. parvum *or *U. urealyticum *was limited, and has been considered less serious in urogenital infections [56-58]. At present, we do not have any explanation to resolve this contradiction. It is possibly due to the particular experimental design, including detection methods such as PCR or serology, or a lack of accurate quality control for DNA extraction. Our study revealed that *U. parvum *had a significant effect on the presence of *C. trachomatis *in the genital tract of healthy women, suggesting that mixed infection is an important factor in bacterial pathogenesis of the genital tract. Meanwhile, whether this observation is limited to Sapporo City remains unknown. In addition, there was no difference of *C. trachomatis *frequency between *U. parvum *[20% (23/117)] and *U. urealyticum *[20% (5/25)] positive samples. This implies that *U. urealyticum *also may have a potential effect on the presence of *C. trachomatis *as well as *U. parvum*.

**Table 2:** Correlation between prevalence of C. trachomatis and ureaplasmas in the genital swabs

Result for C. trachomatis DNA (n)	No. (%) of samples for *U. parvum DNA testing*		p*	No. (%) of samples for *U. urealyticum DNA testing*		*p*	No. (%) of samples for *Ureaplasma total DNA testing*		*p*
						
	Positive	Negative		Positive	Negative		Positive	Negative	
Positive (40)	23 (57.5)	17 (42.5)	0.02	5 (12.5)	35 (87.5)	0.28	26 (65)	14 (35)	0.01
					
Negative (240)	94 (32.5)	146 (53.5)		20 (8.3)	220 (91.7)		107 (44.6)	133 (55.4)	

*Correlation between these frequencies was analyzed by Fisher's exact test

**Figure 3:**
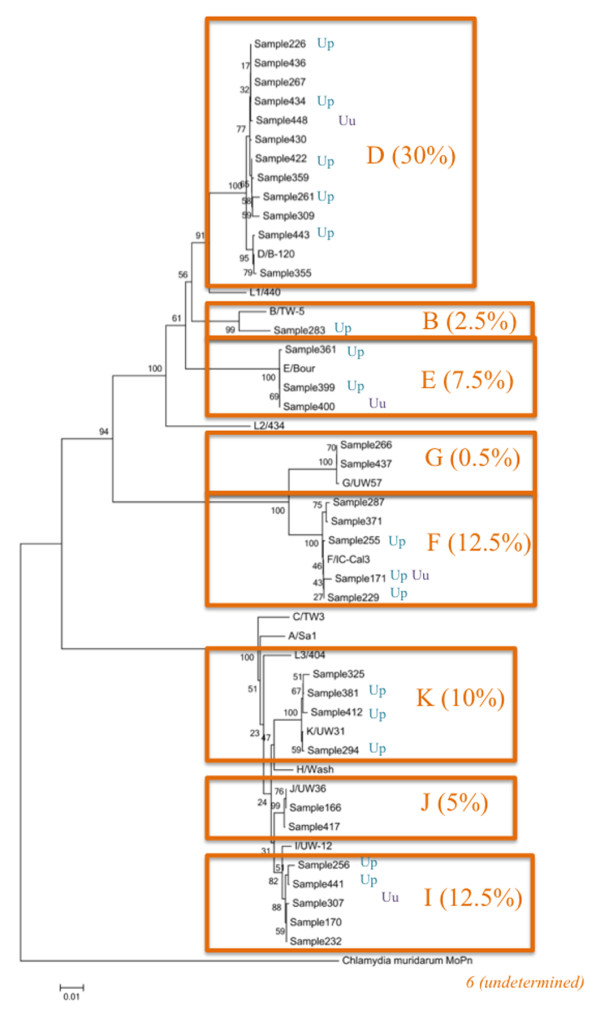
**Phylogenetic trees showing the relationships between *C. trachomatis ompA *PCR amplicons and previously identified bacterial sequences**. Orange letters (B, D-G and I-K), *ompA *genotypes. Parenthetical percentages, the prevalence of each of the *ompA *genotypes. Up (blue letters), *U. parvum*. Uu (purple letters), *U. urealyticum. Undetermined*, these samples failed to amplify *ompA *gene fully because of DNA degradation.

As shown in Figure 3, we also estimated the frequency of *ompA *genotypes among *C. trachomatis *that we detected by direct full sequencing (*n *= 40). As a result, we finally determined the following *C. trachomatis ompA *genotypes: D (30%), B (2.5%), E (7.5%), F (12.5%), G (0.5%), I (12.5%), J (5%), K (10%), and undetermined (17.5%). Although *C. trachomatis ompA *genotype D was the most frequently identified, there was no association of the genotype with either *U. parvum *or *U. urealyticum*.

Why does *C. trachomatis *co-infect with *U. parvum *in the genital tract? So far, we do not have any definite explanation. However, it is possible that the presence of *U. parvum *could provide some advantages for survival of *C. trachomatis *in the genital tract, possibly through directly or indirectly supplying tryptophan to overcome the depletion of this amino acid inside the cells by interferon γ exposure [59,60].

## Conclusion

Endocervical samples were taken from 303 women attending their first prenatal visit at a community hospital in Sapporo, Japan, and the extracted DNAs (*n *= 280), amenable to *16S rRNA *PCR amplification, were analysed by PCR that targeted *C. trachomatis, U. parvum *and *U. urealyticum*. The prevalence of *C. trachomatis, U. parvum *and *U. urealyticum *was 14.3%, 41.7% and 8.9%, respectively. Mixed infection with *C. trachomatis *with either *U. parvum *or *U. urealyticum *was observed in 9.2% of the study population. Interestingly, there was a statistical correlation between the frequency of *C. trachomatis *and either *U. parvum *(*p *= 0.023) or *Ureaplasma *total (*p *= 0.013), but not *U. urealyticum *(*p *= 0.275). Thus, this study demonstrated that the presence of *Ureaplasma *had a significant effect on the presence of *C. trachomatis *in the genital tract of healthy women.

## Competing interests

The authors declare that they have no competing interests.

## Authors' contributions

TY and MM performed the DNA extraction and the PCR. TY and MM performed the biological detection. TY performed the purification of PCR products for DNA sequencing. MM and HY validated the statistical analysis. HY designed this study. KA and KM performed the sample collection. JM and HY supervised the practical work and the data management. HY wrote the manuscript. All authors approved the final manuscript version.

## Pre-publication history

The pre-publication history for this paper can be accessed here:

http://www.biomedcentral.com/1471-2334/12/82/prepub
